# Unilateral Toxic Anterior Segment Syndrome Resulting in Cataract and Urrets-Zavalia Syndrome after Sequential Uneventful Implantation of a Posterior Chamber Phakic Toric Intraocular Lens at Two Different Surgical Facilities: A Series of Unfortunate Events

**DOI:** 10.1155/2020/1216578

**Published:** 2020-11-03

**Authors:** Kepa Balparda, Claudia Marcela Vanegas-Ramirez, Johny Márquez-Tróchez, Tatiana Herrera-Chalarca

**Affiliations:** ^1^Department of Cornea and Refractive Surgery, Black Mammoth Surgical, Medellín, Colombia; ^2^Department of Ophthalmology, Universidad Pontificia Bolivariana, Medellín, Colombia; ^3^Department of Clinical Research, Black Mammoth Surgical, Medellín, Colombia

## Abstract

**Background:**

Phakic Intraocular Lens (P-IOL) implantation is a safe, easy, predictable intervention designed to manage moderate to high refractive errors. Complications are relatively uncommon and include mainly cataract and intraocular pressure spikes. Toxic Anterior Segment Syndrome (TASS) is a rather unusual sterile anterior segment inflammation after uneventful intraocular surgery, extremely rarely reported after P-IOL implantation. Urrets-Zavalia Syndrome (UZS) is also very rarely described after P-IOL. To date, to the best of the authors' knowledge, no article has ever described the simultaneous occurrence of TASS and UZS in a patient after P-IOL implantation.

**Objective:**

In this article, the authors present the case of a female patient with moderate myopic astigmatism, who underwent sequential P-IOL implantation at two different facilities. The postoperative course of the first eye was uneventful, but she developed complications associated to the intervention in the second eye.

**Materials:**

The article describes the case of a young patient who underwent a sequential Phakic Intraocular Lens (P-IOL) implantation at two different institutions. The postoperative course of the first eye (left eye) was uneventful; however, the second eye (right eye) initially developed Toxic Anterior Segment Syndrome (TASS). Although timely and correct management was instituted, upon resolution of TASS, the patient developed Urrets-Zavalia Syndrome, anterior subcapsular cataract, and significant endothelial damage in the same eye.

**Results:**

The patient was followed closely and managed accordingly; corneal edema and anterior segment inflammation of the right eye eventually resolved. Nevertheless, an anterior subcapsular cataract and a fixed dilated pupil remained; with normal intraocular pressure (IOP). Specular microscopy confirmed an endothelial cell loss in the TASS eye (right eye). Pupil size showed no reaction to repeated doses of Pilocarpine 2%. A month after surgery, refraction on her right eye was +0.25 + 0.75 × 93, which resulted in a 20/50 vision.

**Conclusions:**

TASS and UZS are both extremely rare complications after uneventful P-IOL implantation, with only a handful of cases having been reported of each of them. To date, this is the very first case where UZS ensued after and potentially as a consequence of TASS in a patient who had undergone P-IOL implantation. Although a direct causative element could not be pinpointed, the fact that the complication ensued after being operated in one surgical institution and not the other, could suggest some role of different sterilization and handling procedures, but no direct conclusion can be made on this case.

## 1. Introduction

Phakic Intraocular Lens (P-IOL) implantation is a safe, easy, predictable intervention to manage moderate to high refractive errors. Complications are relatively uncommon and include mainly subcapsular anterior cataracts, most of which are not visually significant. Toxic Anterior Segment Syndrome (TASS) is a rather unusual sterile anterior segment inflammation after uneventful intraocular surgery, very rarely reported after P-IOL implantation. Urrets-Zavalia Syndrome (UZS) is defined as a fixed dilated pupil, initially described after penetrating keratoplasty in keratoconus patients who had received atropine. Nowadays, it has been related to many other anterior segment surgeries. To date, no article has ever described the simultaneous occurrence of TASS and UZS in a patient after P-IOL implantation.

In this article, the authors present the case of a female patient with moderate myopic astigmatism, who underwent sequential P-IOL implantation at two different facilities. The postoperative course of the first eye was uneventful, but she developed TASS in the second eye. Although timely and correct management was instituted, upon resolution of TASS, the patient developed UZS, anterior subcapsular cataract, and significant endothelial damage.

## 2. Case Report

A female, 26-year-old patient, lawyer by profession, concurred to the main author's (K. B.) private practice in the search for surgical correction of her refractive error. She had a history of soft contact lens use for most of the day, with some mild foreign body sensation at the end of the day. She had no prior ocular surgeries. Otherwise, her clinical history was unremarkable.

Upon clinical evaluation, her uncorrected distance visual acuity (UDVA) was 20/1600 on both eyes. Her subjective refraction was –6.50 + 2.00 × 90 and –7.75 + 1.75 × 80 on her right and left eyes, respectively, and she achieved a 20/20 vision in both eyes with refraction. Anterior segment evaluation was utterly unremarkable. Her posterior segment was evaluated by a Retina Specialist who determined there were no predisposing peripheral lesions.

Preoperatory Pentacam, endothelial cell count, and biometry were all normal. The patient and the main author discussed different refractive options and jointly decided to perform EyeCryl Phakic Toric IOL (Biotech Vision Care; Ahmedabad, India) implantation in order to better preserve corneal biomechanical stability and avoid potential ectatic risks associated with laser surgery. Lenses were calculated using Biotech's proprietary calculator, with a planned refractive target of +0.50 in both eyes. White-to-white distance was measured both by Pentacam and with a caliper at the slit lamp.

**Figure 1:**
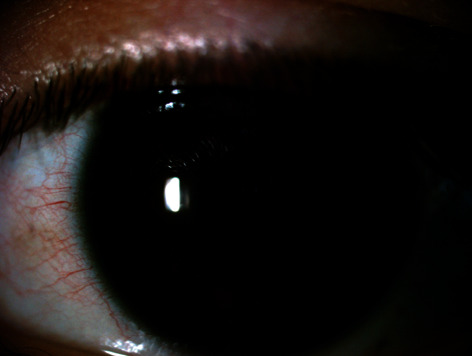
Left eye one week after surgery, with an uneventful clinical course.

The patient was first operated on her left eye at a big multispecialty hospital located in Rionegro (Colombia), and a ‑9.50 + 2.00 × 90 was implanted uneventfully; the lens was left at 80 degrees following calculation. Surgical implantation was performed under peribulbar anesthesia by the main author (K. B.) as follows: the patient was draped and the eye cleaned; then, a 1.2 mm paracentesis and a 2.8 mm main incision were created, and the anterior chamber was filled with 2.4% Sodium Hyaluronate Ophthalmic Viscosurgical Device (Bio-Hyalur HV; Biotech Vision Care; Ahmedabad, India). The P-IOL was mounted and injected inside the anterior chamber. Afterward, the lens was positioned in the correct toric markings, and all four haptics were positioned behind the iris over the ciliary sulcus. Then, the Ophthalmic Viscosurgical Device was removed, and 1% Acetylcholine was injected intracamerally to achieve proper pupillary miosis. The postsurgical regimen for the left eye was Gatifloxacin 0.3% and Prednisolone 1.0% (Zypred; Allergan; Dublin, Ireland). In the first week after surgery, the operated eye was calm, with no pain, a UDVA of 20/15, and refraction of +0.25 + 0.25 × 85 (Figure 1).

The right eye was operated on a month later at a smaller, Ophthalmology-only surgical facility in Medellín (Colombia). The reason for the change was due to the patient's direct preference due to a shorter waiting list in the second facility. The surgical process was exactly the same as with the other eye, using the same brands of fluids and medicines. A –8.00 + 2.50 × 90 EyeCryl Phakic Toric IOL was implanted without complications in a comfortable, uneventful surgery. The lens was left at the 90° meridian. The postsurgical regimen was started with Ciprofloxacin 0.3% and Dexamethasone 0.1% (Flobact-D; Ophtha; Bogotá, Colombia).

**Figure 2:**
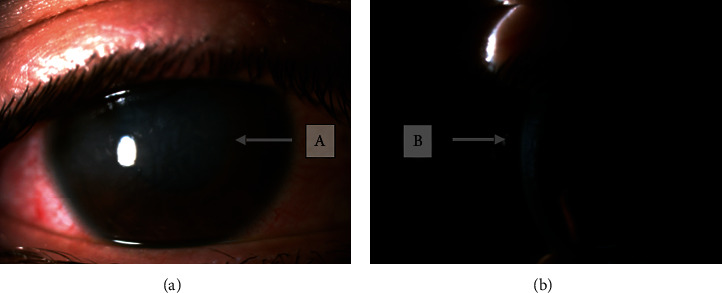
Corneal edema in the right eye during TASS, as seeing both with diffuse (a) and laminar (b) lighting.

The patient requested for an urgent evaluation about 10 hours after surgery, because of exacerbated, uncontrolled pain in her just-operated eye. On clinical evaluation, a grade I corneal edema was found, with a mildly dilated, poorly-reactive pupil and an intraocular pressure of 30 mmHg. She was started on preservative-free Dorzolamide 2%, Timolol Maleate 0.5%, and Brimonidine 0.2% (Krytantek Ofteno PF; Sophia, Mexico) and oral Acetazolamide at a dose of 250 mg every 8 hours. The next day, corneal edema had increased to grade III (Figure 2), with a wider unreactive pupil, with an IOP of 20 mmHg and no pain. A TASS was suspected, so she was instructed to use Prednisolone 1% every hour and keep on using the other medicines.

During the course of four days, she was seen daily, finding a normal, stable IOP of 10 mmHg, but with a constantly edematous cornea and a widely mydriatic, fixed pupil. Pentacam confirmed an edematous cornea, and an anterior segment OCT discarded Descemet's membrane detachment. Due to nonresponsiveness, she was started on oral Prednisolone at a dose of 0.8/mg/kg/day. Infectious endophthalmitis was discarded.

**Figure 3:**
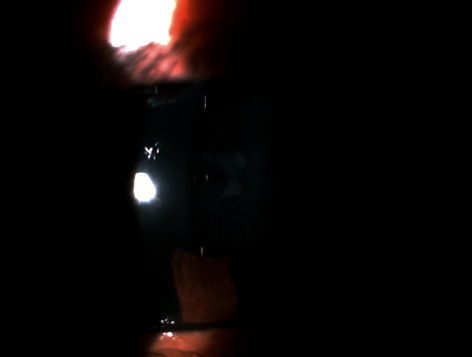
Anterior subcapsular cataract after TASS resolved.

**Figure 4:**
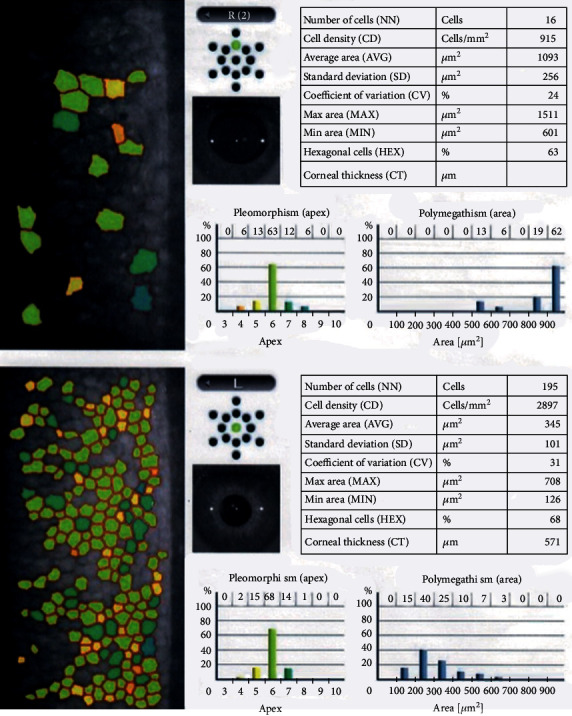
Specular microscopy of both eyes, showing a significant decrease in cell count and a clear increase in cell area in the right eye, while values remain normal in the left eye. Please also note the difference in pupil size between the right (UZS) and left (normal) eyes.

**Figure 5:**
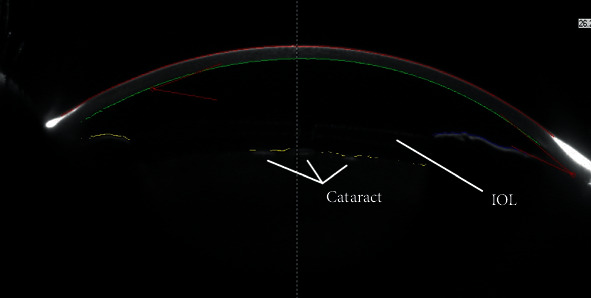
Anterior segment densitometry (Pentacam) demonstrating a correctly implanted intraocular lens (IOL) with a proper vault over the crystalline lens. Anterior subcapsular cataract can also be seen.

**Figure 6:**
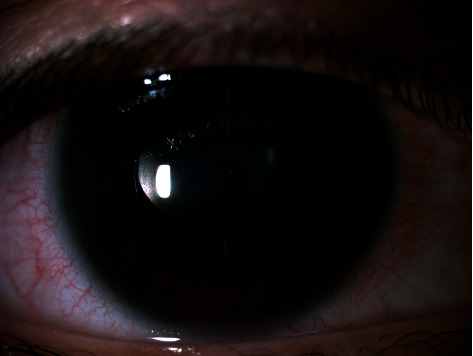
Fixed dilated asymmetric pupil in the right eye, consistent with UZS.

During two weeks, she was followed closely. Corneal edema and anterior segment inflammation eventually resolved, and topical and systemic steroids were eventually tapered. Nevertheless, an anterior subcapsular cataract (Figure 3) and a fixed dilated pupil remained. A month after surgery, refraction on her right eye was +0.25 + 0.75 × 93, which gave her a 20/50 vision. Her IOP has remained normal. Specular microscopy confirmed an endothelial cell loss in the TASS eye, with a normal cell count in the contralateral eye (Figure 4). Pentacam has confirmed the resolution of edema (Figure 5). Pupil size showed absolutely no reaction to repeated doses of Pilocarpine 2% (IsoptoCarpina; Laboratorios Alcon, Colombia) (Figure 6).

Her left eye has remained uneventful, with a stable +0.25 + 0.25 × 82 refraction and a UDVA of 20/15.

**Figure 7:**
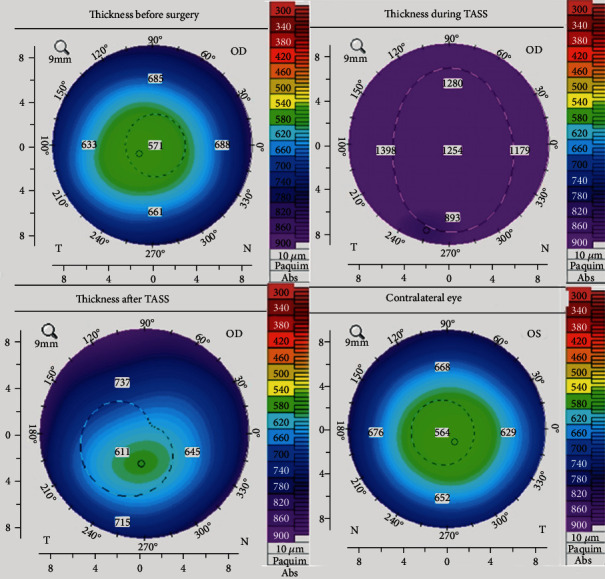
Corneal thickness before surgery, during and after TASS of the right eye. Thickness of contralateral eye.

We attach the corneal thickness before surgery, during and after TASS of the right eye; and the contralateral eye thickness **(**Figure 7).

## 3. Discussion

A myriad of data has confirmed P-IOL to be an effective option for surgical correction of moderate to high myopia and astigmatism, with an excellent safety profile. [1] Yasa et al. [2] published their experience in 58 eyes of 29 patients implanted with the EyeCryl Phakic IOL, finding a great increase in UDVA with stable postoperative refraction after 12 months of following. The group did not find any “significant cataract formation, significant endothelial cell loss, glaucoma, uveitis, or any other vision-threatening complication.” [2] A recent paper by Bianchi also found Implantable Phakic Contact Lens (IPCL V2.0; Care Group, India) implantation to be safe and effective, with stable endothelial cell count and no cases of cataract after 6 months of following. [3]

Long-term following studies have also confirmed these findings. Nakamura et al. [4] retrospectively evaluated results of 114 eyes from 61 patients who underwent Implantable Collamer Lens (ICL; STAAR Surgical; Monrovia, California, United States) implantation at least 10 years before, finding an endothelial cell loss of 5.3% over the years, with only four eyes (3.5%) requiring IOL extraction due to subcapsular cataract. It is to be mentioned that the models of ICL included in this study lacked the central hole currently used, which could theoretically decrease the incidence of symptomatic cataract due to improved aqueous flow in the anterior capsular area [5].

TASS is a very uncommon, but potentially devastating, [6] complication of intraocular surgery, characterized by a severe, sterile postoperative inflammation generally occurring within days of surgery; although, delayed cases have also been reported [7]. Overall, between 3 and 20 cases of TASS occur in the United States of America every year [8]. Sengupta et al. [9] have suggested the incidence of TASS to be around 0.22% in cataract surgeries. Although clusters of TASS have been demonstrated to occur [10], half of the cases are sporadic [9]. By far, most cases of TASS are related to cataract surgery, but some authors have reported its occurrence after keratoplasty [11] and other surgeries.

TASS after P-IOL implantation is considered to be an extremely rare complication, [12] with only a handful of cases being reported so far worldwide [6, 12–14]. Singh et al. [12] reported a case of unilateral TASS in a young patient implanted with ICL, which responded to both oral and topical steroids [12]. Van Philips [6] has published four cases of TASS (two occurring sequentially in one patient) after uneventful implantation of an iris-fixated foldable P-IOL (Artiflex; Ophtec Inc; Groningen, The Netherlands). So far, the biggest case series of TASS after P-IOL implantation is the article published by Hernández-Bogantes et al. [15] that presented the occurrence of six cases of TASS in three patients after same day implantation of ICL. Although no direct causative element could be demonstrated, the authors suggested “the most probable cause [to be] the handling of the ICLs or instrument tips with powdered-gloves as reported by the surgeon” [15].

UZS is another rare complication occurring after uneventful intraocular surgery, initially described after penetrating keratoplasty, but reported after multiple surgeries. It is characterized by a fixed, dilated pupil, with no responsiveness to topical Pilocarpine [16]. Although the exact mechanism for the occurrence of UZS has not been elucidated, [17] “an acute increase in intraocular pressure and ischemia of the iris most probably play a major role” [16]. UZS after P-IOL is extremely rare and has only been reported a couple of times [18, 19]. Arendt and Gerding [20] reported the occurrence of UZS after Artiflex implantation, which developed a pupillary block (IOP 50 mmHg) due to a malfunctioning iridectomy and had to undergo another surgical iridectomy. After the second surgery, the patient developed an unresponsive, fixed, dilated pupil. UZS has also been reported in two patients undergoing uneventful ICL implantation [19].

So far, to the best of the authors' knowledge, no case of associated UZS and TASS secondary to P-IOL has ever been reported in the literature before. In our patient, TASS coursed with an increase in intraocular pressure (30 mmHg) for two days, which may explain the later occurrence of UZS in concordance to the main etiologic theory of iris ischemia secondary to elevated intraocular pressure. Although transient increases in intraocular pressure may be secondary to other causes after P-IOL implantation (such as retained OVD), TASS is directly related to increases in intraocular pressure and seems to be the most plausible culprit in the case presented. After intensive local and systemic steroids, TASS resolved completely, leaving a fixed dilated pupil and other permanent damages, such as anterior subcapsular cataract and endothelial damage. All these changes can be explained by the intense sterile anterior segment inflammation that characterizes TASS.

Although an exact causative agent for the occurrence of TASS cannot be pinpointed in many cases, there is a myriad of potential culprits, including residues of enzymatic detergents in the tip of the instruments, idiopathic response to certain medications, stabilizing agents, and bacterial contamination in the solutions used. It is interesting that the patient developed TASS after being operated in a determined surgical facility, after having had an unremarkable postoperative course when the first eye was operated at another hospital. It may be feasible that different sterilization and handling protocols at the different facilities, as well as the use of different brands of fluids and medications, may have played a role in the appearance of this complication [21]. Nevertheless, the facility where the TASS ensued is an Ophthalmology-only center, with no known outbreaks or previously reported cases of TASS, so it may be reasonable to assume that current sterilization and handling protocols are mainly correct. The change of medication brands may have played a role, and potentially, the patient had an idiopathic response to one of the stabilizing elements used by one brand and not the other.

After a judicious evaluation of the case by the whole surgical and administrative team, no exact, demonstrable causative element for TASS could be determined.

## 4. Conclusion

Literature has demonstrated P-IOL implantation to be “predictable and safe option for refractive correction in highly myopic eyes” [22], with a very low incidence of adverse events. TASS and UZS are both extremely rare complications after uneventful P-IOL implantation, with only a handful of cases having been reported of each of them. To date, this is the very first case where UZS ensued after and potentially as a consequence of TASS in a patient who had undergone P-IOL implantation. Although a direct causative element could not be pinpointed, the fact that the complication ensued after being operated in one surgical facility and not the other could suggest some role of different sterilization and handling procedures, but no direct conclusion can be made on this case.

## Data Availability

All data from this case is available upon request from the lead author (Kepa Balparda).
